# Supplementary material for: A Smo/Gli Multitarget Hedgehog Pathway Inhibitor Impairs Tumor Growth
Source: Cancers (Basel). 2019 Oct 9;11(10):1518. doi: 10.3390/cancers11101518 (PMC6826940; doi:10.3390/cancers11101518)
Supplement: Supplementary file 1 [file cancers-11-01518-s001.pdf]

Supplementary Materials: A Smo/Gli Multitarget Hedgehog Pathway Inhibitor Impairs Tumor Growth

Ludovica Lospinoso Severini, Deborah Quaglio, Irene Basili, Francesca Ghirga, Francesca Bufalieri, Miriam Caimano, Silvia Balducci, Marta Moretti, Isabella Romeo, Elena Loricchio, Marella Maroder, Bruno Botta, Mattia Mori, Paola Infante, Lucia Di Marcotullio

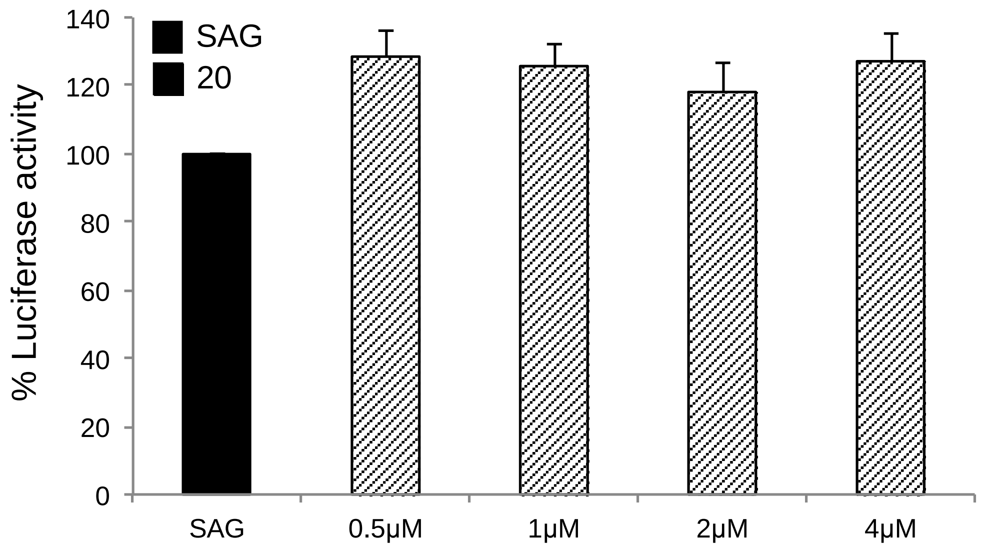

Figure S1. Hh inhibition by compound **20**. Dose-response curve in SAG-treated NIH3T3 Shh-Light II cells. Cells were treated for 48 h with increasing concentrations of compounds **20**. Data were normalized against Renilla luciferase. Data show the mean ± SD of three independent experiments.

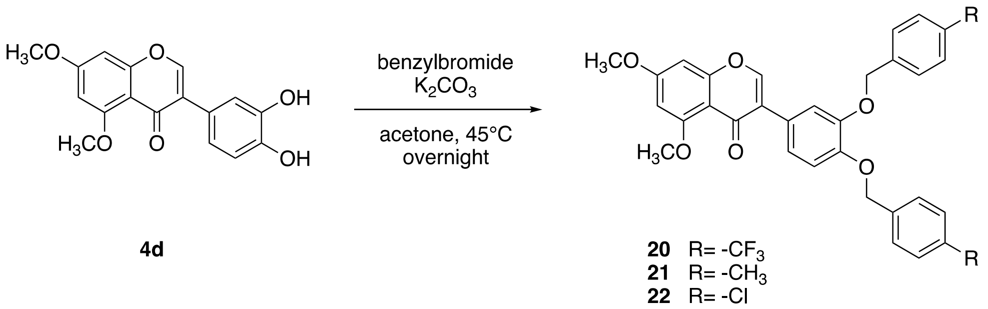

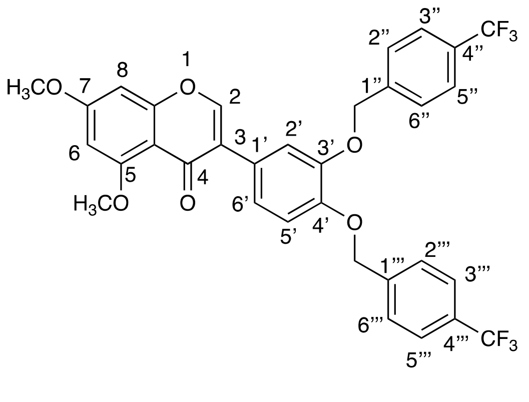

3-(3,4-bis((4-(trifluoromethyl)benzyl)oxy)phenyl)-5,7-dimethoxy-4H-chromen-4-one (**20**)

White solid (Yield 65%) mp: 182-184°C ^{1}H NMR (400 MHz, $CDCl_3$) δ 7.74 (s, 1H, H-2), 7.65-7.52 (m, 8H, H-2′′, H-2′′′, H-3′′, H-3′′′, H-5′′, H-5′′′, H-6′′, H-6′′′), 7.38 (d, *J* = 1.7 Hz, 1H, H-2′), 7.00 (dd, *J* = 8.3, 1.8 Hz, 1H, H-6′), 6.93 (d, *J* = 8.3 Hz, 1H, H-5′), 6.43 (d, *J* = 2.2 Hz, 1H, H-6), 6.37 (d, *J* = 2.2 Hz, 1H, H-8), 5.23 (s, 2H, CH_2), 5.22 (s, 2H, CH_2), 3.95 (s, 3H, OCH_3), 3.89 (s, 3H, OCH_3). ^{13}C NMR (100 MHz, $CDCl_3$): δC 173.42, 164.73, 161.07, 157.91, 149.01, 147.98, 140.93, 132.25, 128.85, 127.68, 125.45, 125.19, 124.94, 122.82, 121.45, 120.38, 116.09, 116.40, 113.40, 109.21, 96.65,94.94,71.53,56.52, 55.96. ESI-MS(*m/z*): $[M+H]^+$calcd. for $C_{33}H_{25}F_6O_6$, 631.1; found, 631.3.

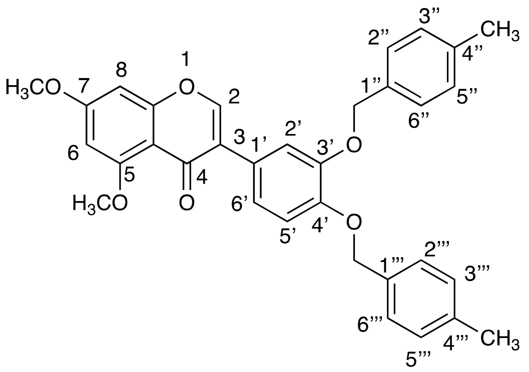

3-(3,4-bis((4-methylbenzyl)oxy)phenyl)-5,7-dimethoxy-4H-chromen-4-one (**21**)

Pale Brown Solid (Yield 70%); mp: 148.0-153.8 °C; ^{1}H NMR (400 MHz, $CDCl_3$): δH 7.70 (s, 1H, H-2), 7.38-7.28 (m, 5H, H-2′, H-2″, H-2″′, H-6″ and H-6″′), 7.16 (d, *J*=7.9 Hz, 4H, H-3″, H-3″′, H-5″, H-5″′),, 6.98 (dd, *J* = 8.3 Hz, *J* = 2.0 Hz, 1H, H-6′), 6.93 (d, *J* = 8.3 Hz, 1H, H-5′), 6.43 (d, *J* = 2.3 Hz, 1H, H-6), 6.37 (d, *J* = 2.3 Hz, 1H, H-8), 5.14 (s, 2H, CH_2), 5.3 (s, 2H, CH_2), 3.94 (s, 3H, OCH_3), 3.88 (s, 3H, OCH_3), 2.35 (s, 6H, CH_3); ^{13}C NMR (100 MHz, $CDCl_3$): δC 175.45, 163.98, 161.60, 159.96, 150.37, 149.07, 148.84, 137.51, 137.48, 134.47, 129.24, 129.20, 127.71, 127.49, 125.97, 125.55, 122.14, 116.50, 115.02, 110.06, 96.31, 92.64, 71.39, 71.33, 56.52, 55.85, 21.34. ESI-MS (m/z): $[M+H]^+$calcd. for $C_{33}H_{31}O_6$, 523.59; found, 523.60

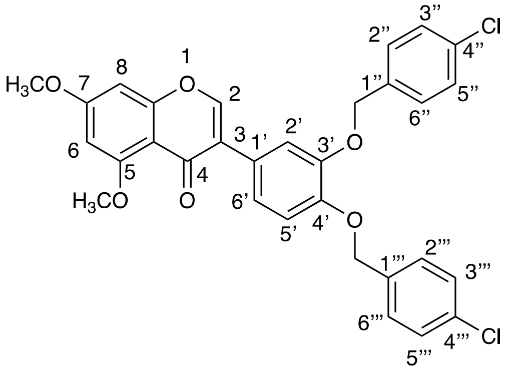

3-(3,4-bis((4-chlorobenzyl)oxy)phenyl)-5,7-dimethoxy-4H-chromen-4-one (**21**)

Pale Yellow Solid (Yield 90%); mp: 158.0-160.0 °C; ^{1}H NMR (400 MHz, $CDCl_3$): δH 7.73 (s, 1H, H-2), 7.42-7.29 (m, 9H, H-2′, H-2″, H-2″′, H-3″, H-3″′, H-5″, H-5″′, H-6″ and H-6″′), 6.98 (dd, *J* = 8.0 Hz, *J* = 1.2 Hz, 1H, H-6′), 6.93 (d, *J* = 8.3 Hz, 1H, H-5′), 6.44 (d, *J* = 2.0 Hz, 1H, H-6), 6.37 (d, *J* = 2.0 Hz, 1H, H-8), 5.13 (s, 2H, CH_2), 5.11 (s, 2H, CH_2), 3.94 (s, 3H, OCH_3), 3.89 (s, 3H, OCH_3); ^{13}C NMR (101 MHz, $CDCl_3$) δ 175.41, 164.09, 161.62, 159.99, 150.45, 148.71, 148.54, 135.90, 135.88, 133.73, 133.70, 128.93, 128.79, 128.75, 126.04, 125.77, 122.27, 116.61, 114.99, 110.04, 96.40, 92.72, 70.75, 70.73, 56.57, 55.88. ESI-MS(m/z): $[M+H]^+$calcd. for$C_{31}H_{25}Cl_2O_6$, 563.42; found, 563.43

Compound 20

^{1}H NMR (400 MHz, $CDCl_3$)

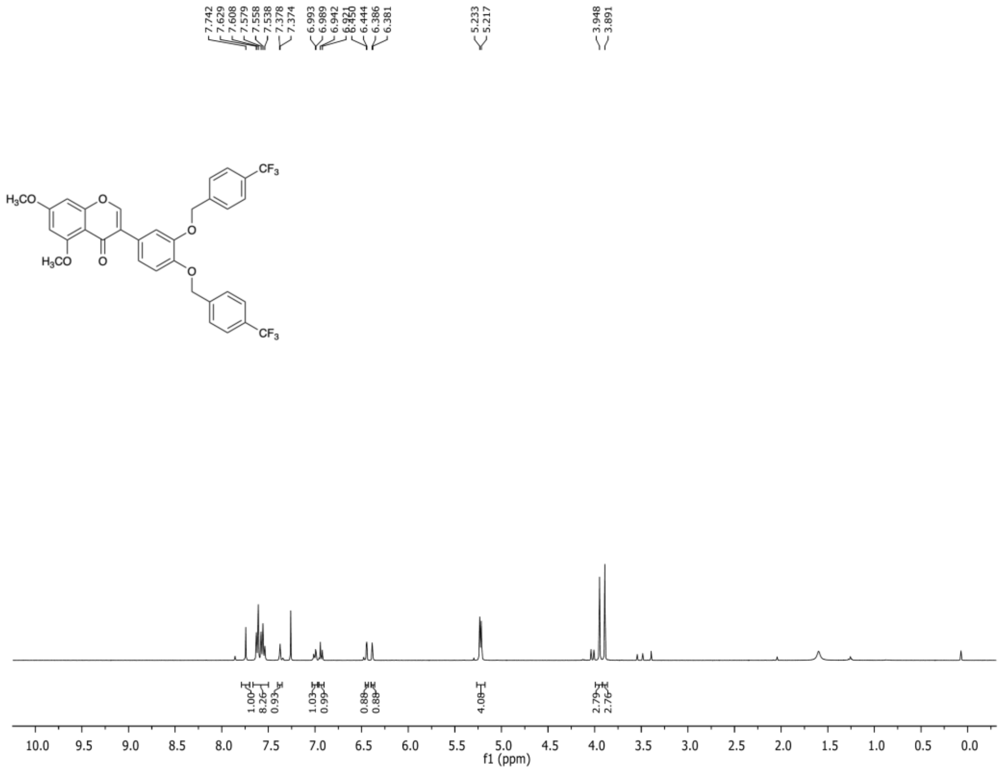

Compound 21

^{1}H NMR (400 MHz, $CDCl_3$)

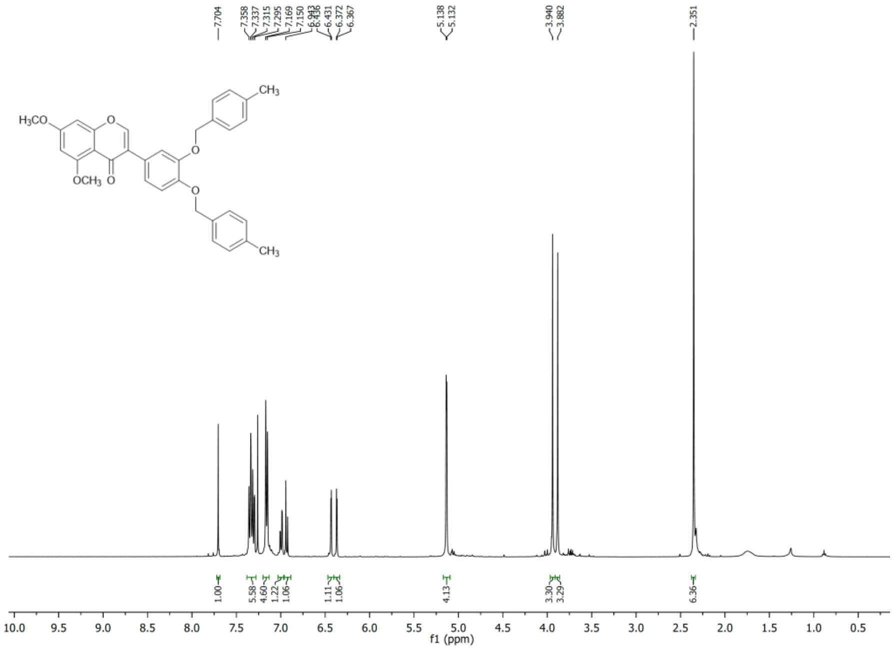

Compound 22

^{1}H NMR (400 MHz, $CDCl_3$)

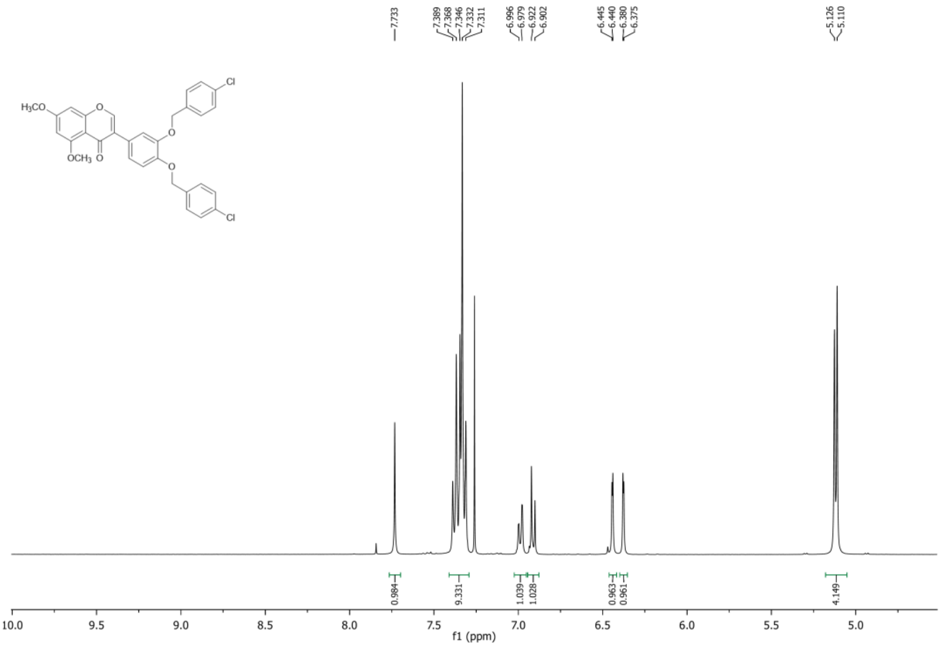

Figure S2. General procedure for the synthesis of compounds 20-22. Characterization of compounds 20-22.

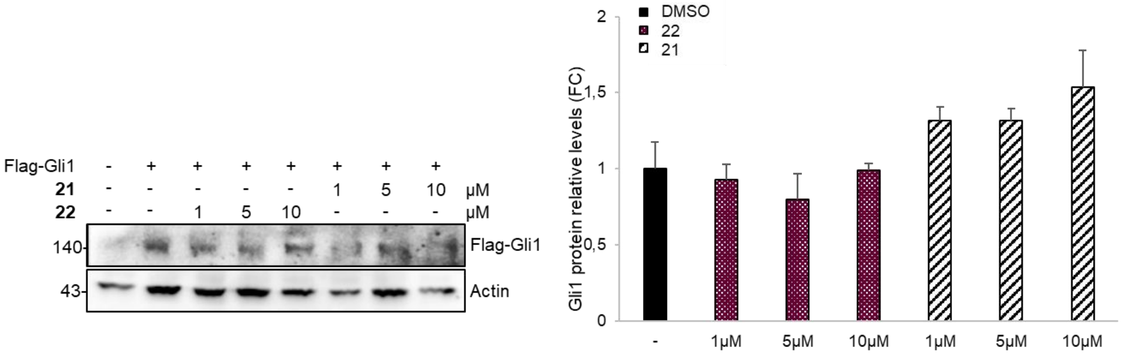

Figure S3. Effect of compounds **21** and **22** on overexpressed Gli1 protein. MEFs WT were transfected with Flag-tagged Gli1 or empty vector, then treated with DMSO only or increasing concentrations of compounds **21** and **22**. The graph shows densitometric analysis ± S.D.

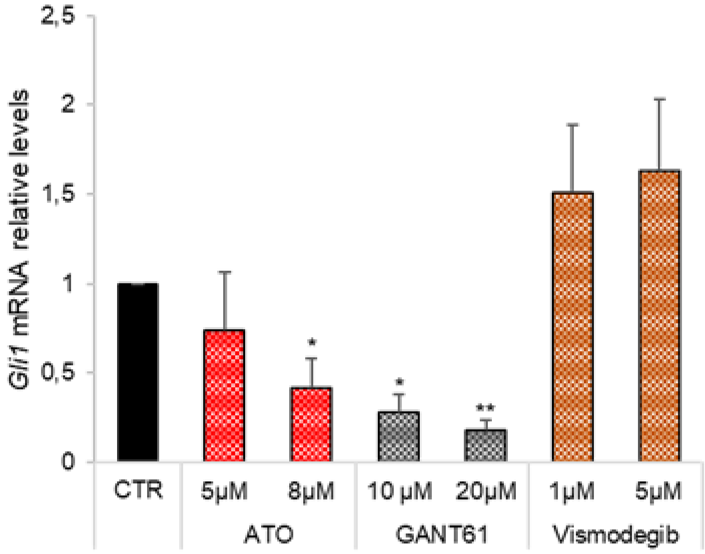

Figure S4. Effect of ATO, GANT61 and Vismodegib on *Gli1* mRNA levels in Smo$^{-/-}$ MEFs. Data show means ± S.D of three independent experiments. (*) $p < 0.05$, (**) $p < 0.01$ vs. CTR.

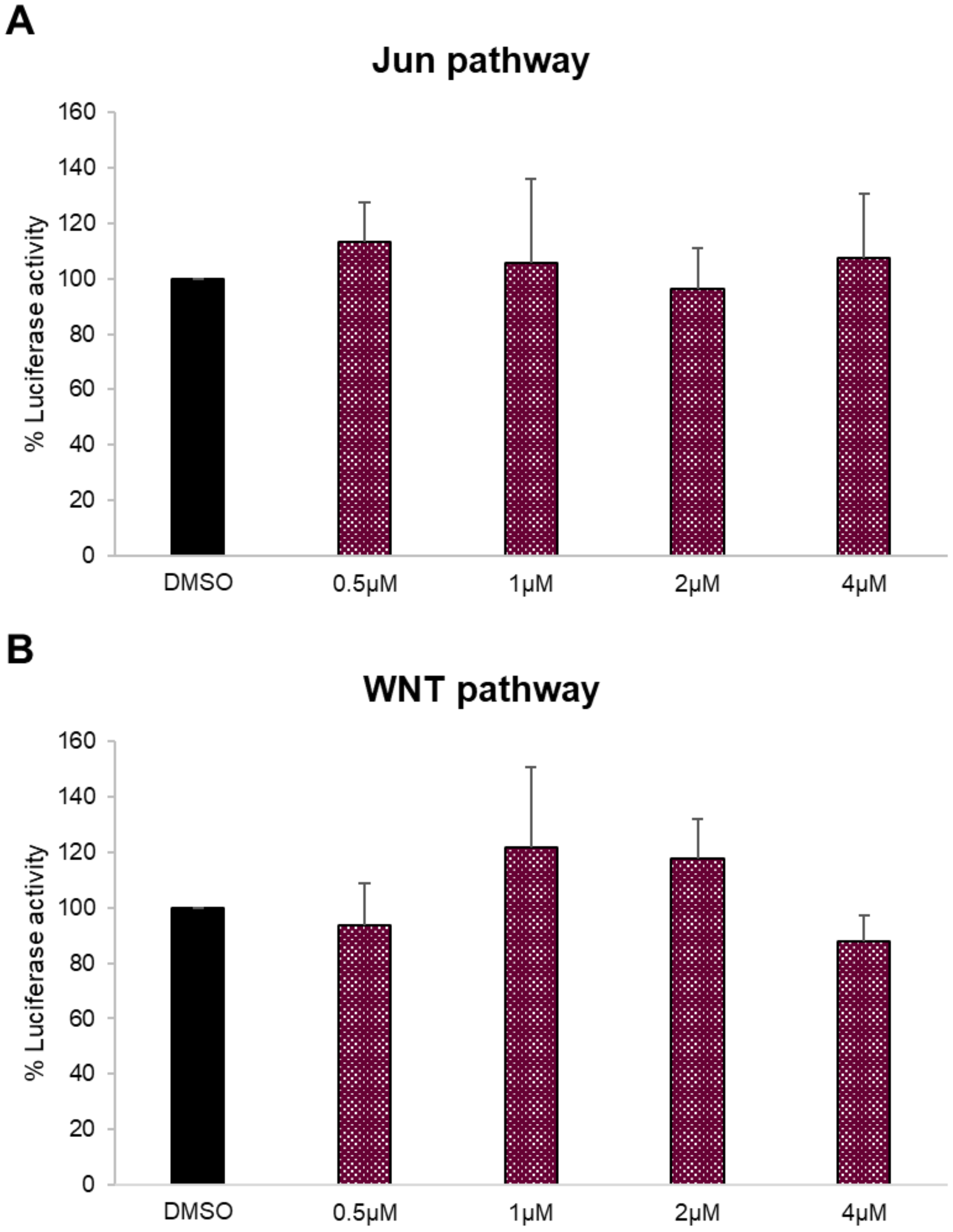

Figure S5. Compound **22** does not affect Jun and WNT pathways. Luciferase assays were performed in MEFs WT co-transfected with MMP1- or TopFlash-luciferase reporter and c-Jun or β-catenin to assay Jun and WNT pathway activity, respectively. 24 h after transfection cells were treated with DMSO only or increasing concentrations of compound **22**. Specific luciferase activities were normalized to Renilla luciferase reporter. Data show means ± S.D of three independent experiments.

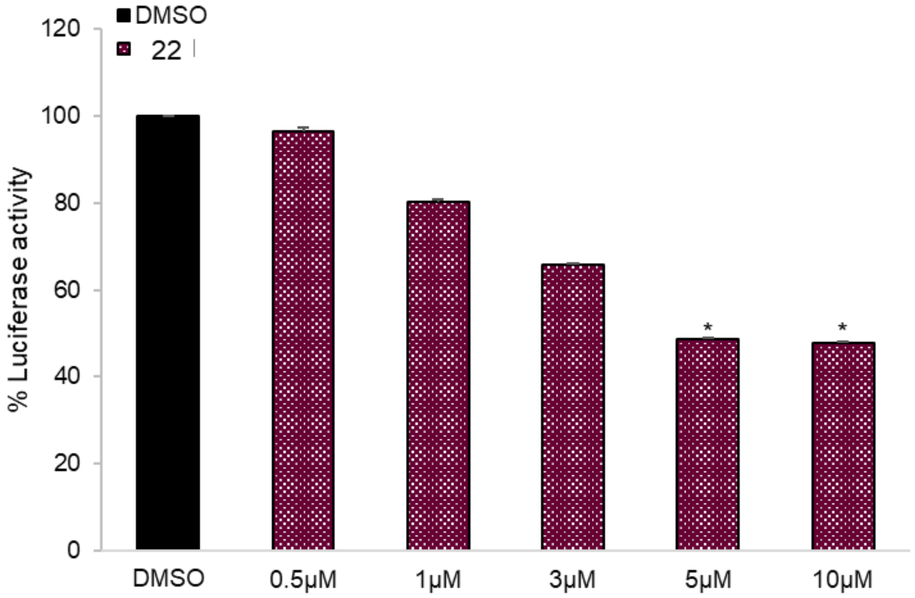

Figure S6. Inhibition of Gli2-induced transcription in MEFs WT treated with increasing concentrations of compound **22**. Luciferase assay was performed in MEFs WT transfected with 12XGliBS-Luc and pRL-TK Renilla (as normalization control) plus empty vector or Gli2 WT; 24 h after transfection cells were treated with DMSO only or increasing concentrations of compound **22**. Luciferase activity was analyzed 24 h after treatment. Data show the mean ± SD of three independent experiments. (*) $p < 0.05$ vs. DMSO.

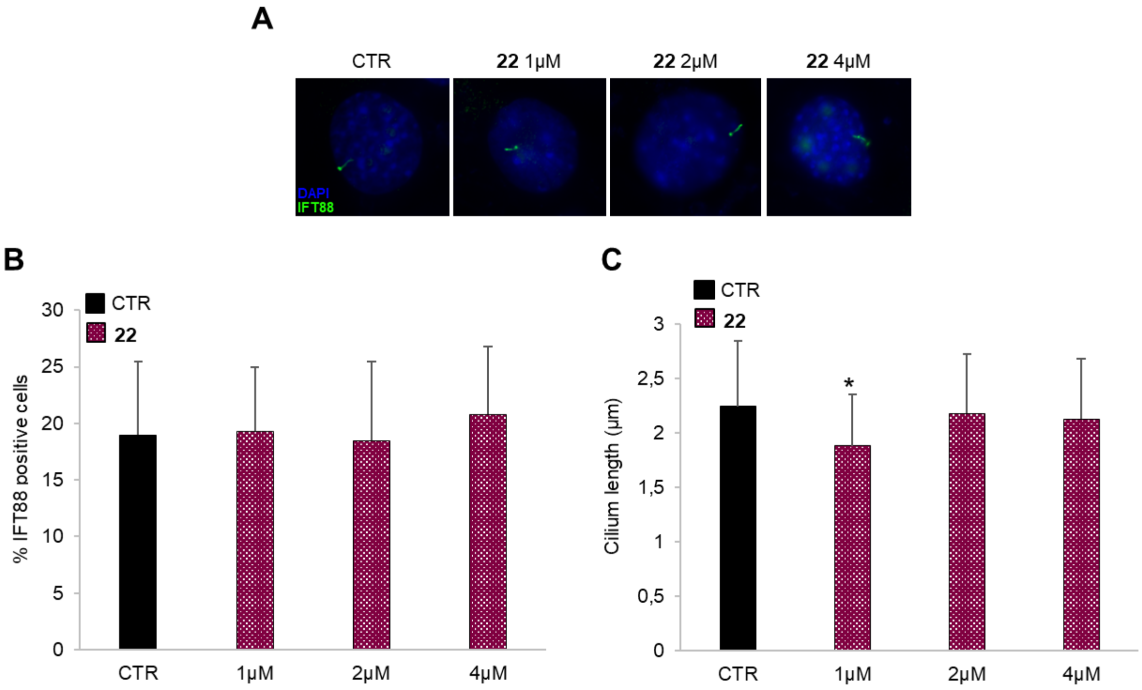

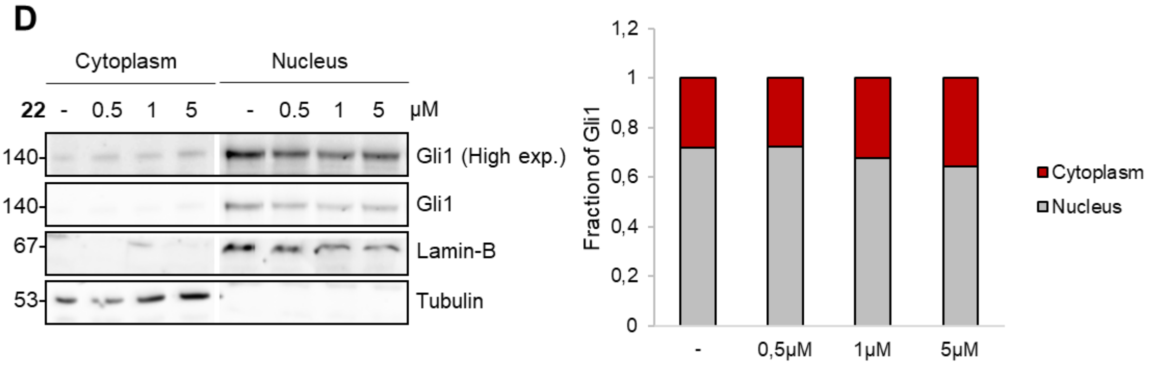

Figure S7. Effect of compound **22** on ciliogenesis and Gli1 localization. (**A-C**) Compound **22** does not affect ciliogenesis. MEFs WT were incubated in the presence or absence of **22** at different concentrations for 24 h, as indicated. Cells were stained for the cilium marker IFT88 and DAPI (**A**), and the number (**B**) and length of cilia (**C**) were quantified from immunofluorescence images. Data shown mean ± S.D. of three independent experiments. (*) $p < 0.05$ vs. CTR. (**D**) Compound **22** does not affect Gli1 subcellular localization. Subcellular fractions were obtained from murine Med-1 MB cells treated with DMSO or increasing concentrations of compound **22**. Lamin-B and Tubulin were used as nuclear and cytoplasmic markers, respectively. The fraction of Gli1 in the cytoplasm or nucleus for each concentration is plotted on the right.

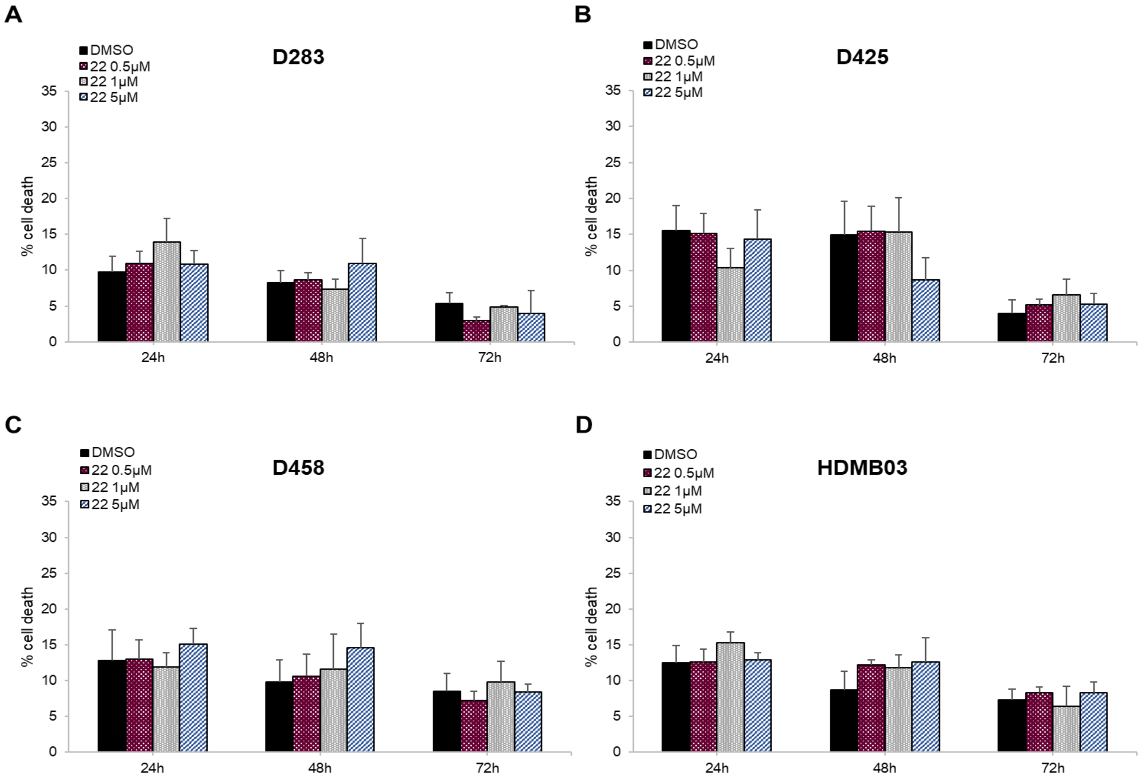

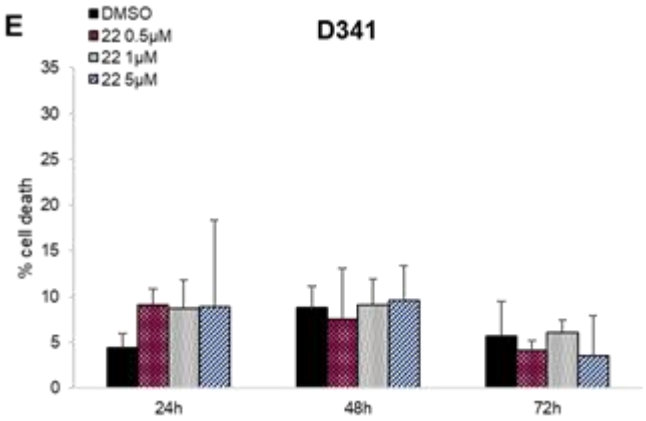

Figure S8. Effect of compound **22** on Hedgehog-independent MB cell lines. MB cells belonging to Group3 (D425, D458, HDMB03, D341) and Gropu3/4 (D283) were treated with DMSO only or increasing concentrations of compound **22**. A trypan blue count was performed after 24–48–72 h of treatment to determine the cytotoxic effects of compound **22**. Data shown mean ± S.D. of three independent experiments.

Table S1. Effect of compounds **21** and **22** on *Gli1* mRNA levels in Smo$^{-/-}$ MEFs. Fold change ($2^{-\Delta Ct}$) is normalized to *Hprt* gene expression.

Treatment	Gli1 Ct Mean	ΔCt Mean	Mean Fold change
DMSO	28.82	7.91	1 ± S. D.
**21** 1 μM	29.22	8.55	0.68 ± S. D.
**21** 2 μM	29.64	8.74	0.58 ± S. D.
**22** 1 μM	29.42	8.51	0.67 ± S. D.
**22** 2 μM	30.34	9.33	0.37 ± S. D.

© 2019 by the authors. Licensee MDPI, Basel, Switzerland. This article is an open access article distributed under the terms and conditions of the Creative Commons Attribution (CC BY) license (http://creativecommons.org/licenses/by/4.0/).
